# Generative AI and large language models in nuclear medicine: current status and future prospects

**DOI:** 10.1007/s12149-024-01981-x

**Published:** 2024-09-25

**Authors:** Kenji Hirata, Yusuke Matsui, Akira Yamada, Tomoyuki Fujioka, Masahiro Yanagawa, Takeshi Nakaura, Rintaro Ito, Daiju Ueda, Shohei Fujita, Fuminari Tatsugami, Yasutaka Fushimi, Takahiro Tsuboyama, Koji Kamagata, Taiki Nozaki, Noriyuki Fujima, Mariko Kawamura, Shinji Naganawa

**Affiliations:** 1 Department of Diagnostic Imaging, Graduate School of Medicine, Hokkaido University, Kita 15, Nishi 7, Kita-Ku, Sapporo, Hokkaido, 060-8638 Japan; 2 Department of Radiology, Faculty of Medicine, Dentistry and Pharmaceutical Sciences, Okayama University, Kita-Ku, Okayama, Japan; 3 Medical Data Science Course, Shinshu University School of Medicine, Matsumoto, Nagano Japan; 4 Department of Diagnostic Radiology, Tokyo Medical and Dental University, Bunkyo-Ku, Tokyo, Japan; 5 Department of Radiology, Osaka University Graduate School of Medicine, Suita-City, Osaka, Japan; 6 Department of Diagnostic Radiology, Kumamoto University Graduate School of Medicine, Chuo-Ku, Kumamoto, Japan; 7 Department of Radiology, Nagoya University Graduate School of Medicine, Showa-Ku, Nagoya, Japan; 8 Department of Artificial Intelligence, Graduate School of Medicine, Osaka Metropolitan University, Abeno-Ku, Osaka, Japan; 9 Department of Radiology, Graduate School of Medicine and Faculty of Medicine, The University of Tokyo, Bunkyo-Ku, Tokyo, Japan; 10 Department of Diagnostic Radiology, Hiroshima University, Minami-Ku, Hiroshima, Japan; 11 Department of Diagnostic Imaging and Nuclear Medicine, Kyoto University Graduate School of Medicine, Sakyoku, Kyoto Japan; 12 Department of Radiology, Kobe University Graduate School of Medicine, Chuo-Ku, Kobe, Japan; 13 Department of Radiology, Juntendo University Graduate School of Medicine, Bunkyo-Ku, Tokyo, Japan; 14 Department of Radiology, Keio University School of Medicine, Shinjuku-Ku, Tokyo, Japan; 15 Department of Diagnostic and Interventional Radiology, Hokkaido University Hospital, Kita-Ku, Sapporo, Japan

**Keywords:** Generative AI, Large language model, Report generation, Report structuring, Education, Nuclear medicine, PET, SPECT

## Abstract

This review explores the potential applications of Large Language Models (LLMs) in nuclear medicine, especially nuclear medicine examinations such as PET and SPECT, reviewing recent advancements in both fields. Despite the rapid adoption of LLMs in various medical specialties, their integration into nuclear medicine has not yet been sufficiently explored. We first discuss the latest developments in nuclear medicine, including new radiopharmaceuticals, imaging techniques, and clinical applications. We then analyze how LLMs are being utilized in radiology, particularly in report generation, image interpretation, and medical education. We highlight the potential of LLMs to enhance nuclear medicine practices, such as improving report structuring, assisting in diagnosis, and facilitating research. However, challenges remain, including the need for improved reliability, explainability, and bias reduction in LLMs. The review also addresses the ethical considerations and potential limitations of AI in healthcare. In conclusion, LLMs have significant potential to transform existing frameworks in nuclear medicine, making it a critical area for future research and development.

## Introduction

Despite being systems designed to learn from text, Large Language Models (LLMs) have seemingly acquired a form of intelligence. Since the release of ChatGPT in November 2022, LLMs have rapidly found themselves applied in various fields. A staggering number of papers are being published on a daily basis. In the medical field, they have proven to be incredibly useful, with numerous examples demonstrating their impact. Even a simple search on PubMed using the term "large language model" reveals that 1,000 and 2,000 academic papers that are related to large language models have been published annually in 2022 and 2023, respectively; and as of August 2024, the number has already surpassed 2,400. This equates to about 10 papers on large language models being published every day. Many applications of LLMs have also been proposed in the field of radiology. Our group, TOP GUN, has extensively discussed both the advantages and disadvantages of using LLMs in radiology [1]. Among the possibilities of various applications, radiology report generation stands out as a particularly interesting and highly relevant area [1–5]. Alternatively, LLMs can be utilized to read radiology reports and classify them objectively based on diagnostic criteria like BI-RADS [6].

It has also been demonstrated that LLMs are capable of generating highly effective abstracts for scientific papers [7], and they have even become so adept at writing entire papers that it is difficult to distinguish them from those written by humans [8]. They are also excellent at extracting structured data, and can be conveniently used for providing information to patients. Numerous examples of applications of LLMs mentioned above have been reported across various fields. However, the question of how exactly LLMs can be useful in nuclear medicine is not straightforward. While it is believed that they could be highly useful in nuclear medicine, there has not been much comprehensive review on this subject. Although Alberts and colleagues have discussed their potential applications and pointed out several issues in the European Journal of Nuclear Medicine and Molecular Imaging, further investigations and discussions are needed [9]. In particular, it remains unclear how useful ChatGPT could be in assisting nuclear medicine researchers with writing or summarizing existing nuclear medicine-related texts. The reliability of LLMs in generating contents related to nuclear medicine and molecular imaging has not been sufficiently verified.

In this review, we aim to explore how LLMs can be utilized in nuclear medicine. We will start by discussing the latest advancements in nuclear medicine, and then proceed to analyze how LLMs are being applied in other areas of radiology. Building on these insights, we will consider potential applications in nuclear medicine.

## The latest advancements in nuclear medicine

A nationwide survey, conducted every 5 years, has revealed that in Japan, traditional radiopharmaceuticals continue to be widely used, while several new agents have also been introduced into clinical practice [10]. Notably, among those new agents, the therapeutic radiopharmaceutical [^177^Lu]DOTA-TATE has made significant strides in recent years. Our review last year [11] highlighted the growing body of evidence, which has expanded considerably over the past year, on account of dedicated researchers and clinicians in the field of nuclear medicine. The versatility of nuclear medicine is underscored by its applicability to a wide range of conditions, including inflammation and malignant diseases, its quantitative capabilities, and the use of various tracers beyond 2-[fluorine-18]fluoro-2-deoxy-d-glucose (FDG). These advancements have collectively enhanced the diagnostic and therapeutic potential of nuclear medicine, making it an increasingly valuable tool in modern healthcare.

Examples of tracers beyond FDG include fibroblast activation protein inhibitors (FAPI), prostate-specific membrane antigen (PSMA), fluoromisonidazole (FMISO), and methionine. Research has demonstrated the utility of [^18^F]AlF-NOTA-FAPI-04 in assessing liver fibrosis in patients who underwent liver transplantation [12]. [^18^F]FMISO is a relatively older tracer compared to FAPI and PSMA, but it remains valuable for detecting hypoxia and is also capable of identifying isocitrate dehydrogenase (IDH) mutations in gliomas [13, 14]. [^11^C]Methionine is well known for its effectiveness in brain tumor imaging, and it has also proven to be useful in imaging oral squamous cell carcinoma (SCC) [15]. Despite being an established technology, collimators remain critical components in nuclear medicine, with ongoing efforts to optimize their performance. For instance, Saed and colleagues have sought to characterize the collimator–detector response function of a breast-dedicated SPECT system equipped with a lofthole collimator using GATE Monte Carlo simulations [16].

**Fig. 1:**
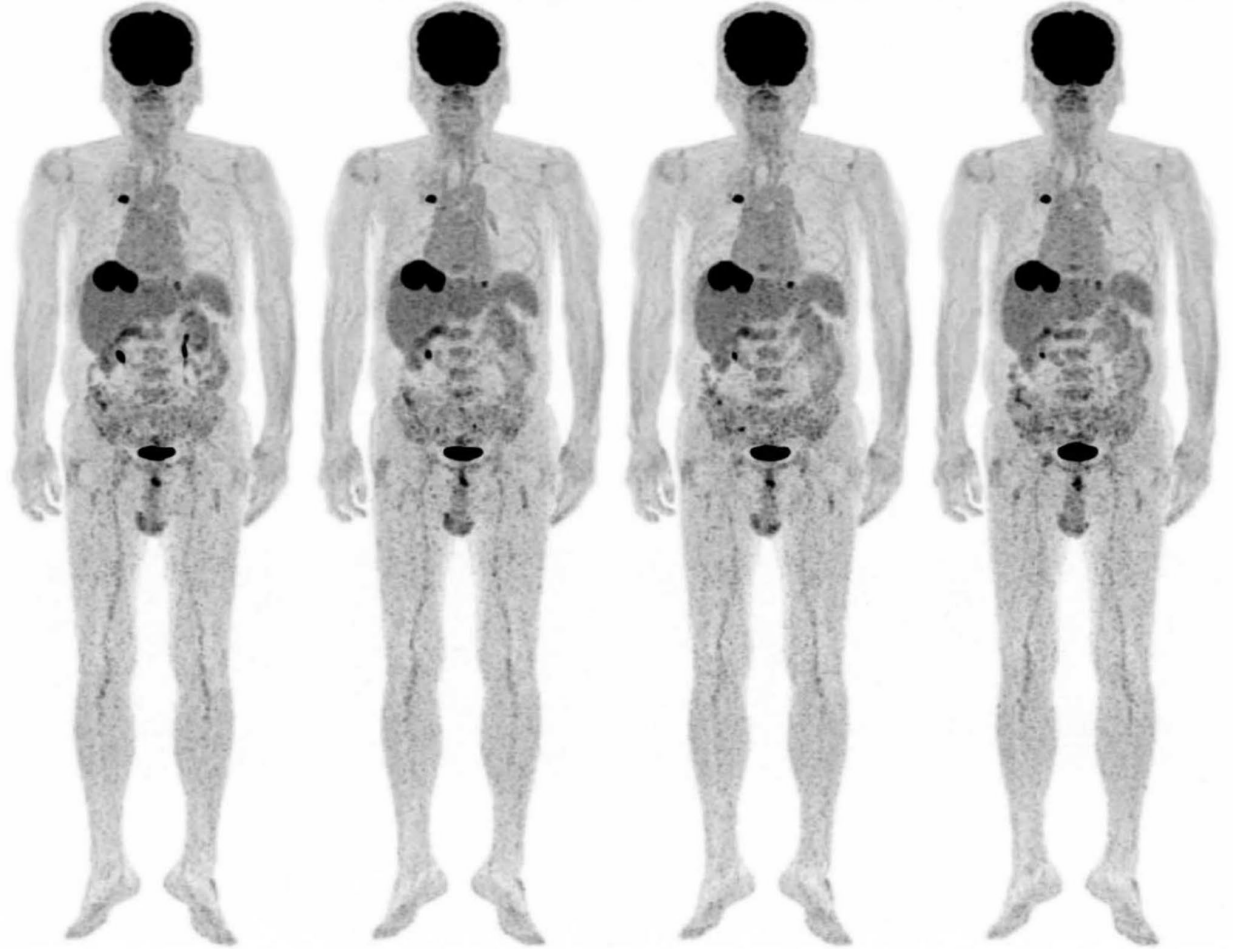
Adapted from Tamaki et al. 2023 [22]. Sequential whole-body FDG-PET scans (3 min each) performed about 60 min post-FDG injection in a patient with lung cancer and pulmonary metastatic lesions. The scans demonstrated strong and sustained uptake in both the primary lung cancer and liver metastases, while uptake changes due to motion were observed in the ureter and small intestine. This distinction helps in differentiating between pathological and non-pathological abdominal accumulations

**Fig. 2:**
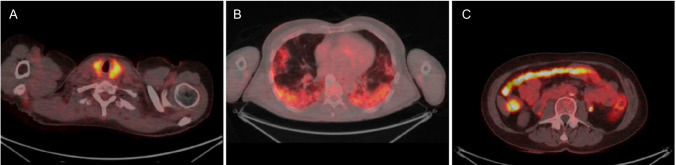
Adapted and modified from Gideonse et al. 2024 [30]. The FDG-PET/CT images of three patients who were diagnosed with immune-related adverse events (irAE) following immunotherapy. **A** Thyroiditis, **B** pneumonitis, and **C** colitis

Other recently discussed tumors and tumor-like diseases where nuclear medicine can be applied include peripheral T-cell lymphoma [17], Castleman disease [18], pulmonary hydatid cyst [19], and recurrent gallbladder cancer [20]. PET morphology is also valuable for characterizing lung nodules as either lung cancer or tuberculosis [21]. Dynamic imaging (4D PET) provides a wealth of clinically useful information, such as distinguishing between pathological and physiological accumulation in the abdomen (Fig. 1), and another example of use of 4D PET is motion correction [22, 23]. In lung cancer, a new parameter called "intratumor metabolic and heterogeneity factor" was tested, and it has been reported to be useful in predicting epidermal growth factor receptor (EGFR) mutations [24]. Understanding normal findings is crucial for accurately identifying pathological conditions, and the atlas of physiological accumulation is useful for that purpose [25]. Knowledge of physiological FDG uptake in neck muscles after neck surgery is also important [26]. Although the accumulation of FDG in both cancer and inflammation presents a limitation, it is advantageous in diagnosing fever of unknown origin [27]. Moreover, PET is useful in evaluating immune checkpoint therapy for malignant melanoma [28]. As immune checkpoint inhibitors are more widely used, information on PD-1/PD-L1 expression has become increasingly significant. PET/CT is a non-invasive method used for detecting PD-1/PD-L1 expression and offers several advantages over immunohistochemistry (IHC). PET/CT can dynamically reflect the expression of PD-1/PD-L1 within the tumor, providing valuable guidance for clinical treatment [29]. Additionally, PET has been reported to be useful in detecting immune-related adverse events (irAEs, Fig. 2) [30]. On the other hand, maximum standardized uptake value (SUVmax) of [^123^I]metaiodobenzylguanidine (MIBG) SPECT may be effective for assessing cardiac function in pheochromocytoma [31]. SPECT/CT has also shown utility in monitoring treatment responses in medication-related osteonecrosis of the jaw (MRONJ) [32]. A meta-analysis indicated that FDG-PET/MRI is useful in detecting lymph-node metastasis in non-small cell lung cancer [33]. Other studies include researches on predicting external radiation doses of [^177^Lu]DOTA-TATE based on tumor size and renal function [34], combining [^99m^Tc]galactosyl human serum albumin (GSA) and indocyanine green (ICG) for preventing posthepatectomy liver failure [35], and assessing renal function simultaneously while evaluating renal tumors with FDG-PET/CT [36].

Nuclear medicine plays a crucial role in dementia as well. In recent years, Amyloid PET has been widely performed, and with the introduction of disease-modifying drugs (Lecanemab) for Alzheimer's disease (AD) in Japan starting in 2024, the number of cases utilizing this technology is increasing. While cerebral blood flow imaging may receive less attention compared to amyloid PET, it is crucial for the precise classification of diseases that amyloid PET cannot distinguish [37]. Differentiating between mild cognitive impairment (MCI) and AD is clinically important, but the presence of brain atrophy can sometimes affect the accuracy of image interpretation. New approaches have been devised to address this issue [38]. Additionally, PET is used in research on other brain conditions, such as attention-deficit hyperactivity disorder (ADHD). Although disturbances in dopamine, serotonin, and norepinephrine functions have been implicated in patients with ADHD, no characteristic findings have been identified from PET studies [39].

In cardiology, nuclear medicine has also proven beneficial. There have been reports showing a decrease in pyrophosphate (PYP) uptake after tafamidis therapy in transthyretin cardiac amyloidosis [40]. Additionally, advancements have been made in the quantification techniques for PYP-SPECT in cardiac amyloidosis [41, 42].

Speaking of quantification, there is ongoing debate about the optimal threshold values for metabolic tumor volume (MTV) and total lesion glycolysis (TLG) in FDG-PET/CT [43]. Another study suggested that the SUVmean ratio of [^13^N]ammonia PET between liver and lung muscles can predict survival after coronary artery bypass grafting (CABG) [44]. Meanwhile, there is a movement to apply the Deauville criteria, a well-established qualitative method for assessing treatment response in malignant lymphoma, to other diseases such as lung cancer [45]. The debate over whether qualitative or quantitative methods are superior is likely to continue in the future.

In CT imaging, ultra-low-dose techniques are being realized with the support of deep learning [46]. Similar advancements are being pursued in PET, with active studies performed by focusing on dedicated breast PET systems that aim to reduce the radiation exposure [47]. Efforts to lower the dose in PET imaging for lung cancer using deep learning are also ongoing [48], and the same applies to colon CT [49]. In terms of diagnostic assistance, there is ongoing work on anomaly detection in chest PET [50]. Regarding domain transformation, one approach involves converting CT images into ventilation imaging [51]. While transforming images into other images is straightforward, converting images into text may seem challenging for humans, but it is not necessarily so for AI. Radiomics remains an active area of research [52, 53]. Several studies have shown that radiomics is highly promising; however, there are critical issues that need to be addressed, including poor standardization and generalization of radiomics results, data quality control, reproducibility, and concerns over model overfitting.

## LLM and radiology reports

Extended models of LLMs, which can process images to return text or images, are referred to as vision-language models (VLMs) [54]. Research on AI that can generate reports directly from images is still in its infancy and has a long way to go. Most studies involve models that take text as both input and output. Although it is still rare in the field of nuclear medicine, there has been exploration into generating conclusions, diagnoses, or impressions of radiology reports from findings [55]. Tie et al. used 12 fine-tuned open-source LLMs to experiment on 37,370 reports, evaluating whether the impressions could be appropriately generated. The results showed that 89% of the cases were judged to be clinically acceptable. There have also been promising attempts to summarize knee MRI reports and cardiac MRI reports [56, 57].

**Fig. 3:**
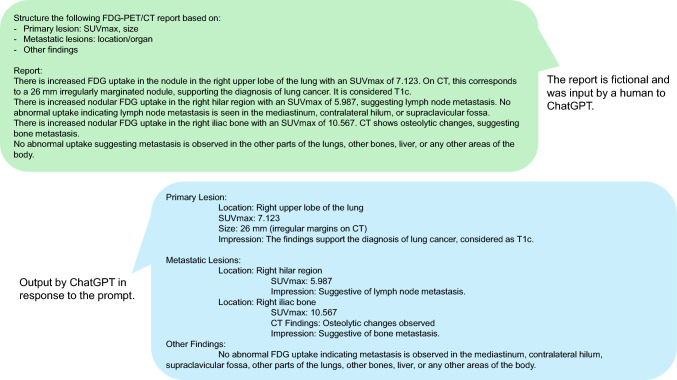
Structuring FDG-PET/CT report using LLM. This experiment, conducted with ChatGPT-4o on August 31st, demonstrates the process of structuring reports using LLMs. Similarly, it is also possible to convert structured reports into regular text

Another interesting example of application is using LLMs to evaluate how senior doctors revise reports created by residents and then using this feedback for resident training [58]. There is also research aimed at improving the readability of orthopedic reports to enhance patient understanding [59]. In this study, Butler et al. reported an overall improvement in readability, but it is important to note that hallucinations occurred in 3–6% of the cases. LLMs are well suited for structuring free-text reports (Fig. 3), and several studies have explored this use. For instance, Ahyad et al. examined the time-saving effects of AI in this context [60]. Their findings showed approximately a 20% reduction in time for structuring reports written in natural language. Additionally, while still not yet perfect, it has been demonstrated that LLMs can translate reports from English into Spanish, Hindi, and Russian [61]. Though it is not an AI study, there was research on verifying missed diagnoses by including the phrase “visible in retrospect” in subsequent reports [62]. For radiologists to flag such cases proactively, this rule would need to be implemented in the radiology department, but with LLMs, it might be possible to detect “visible in retrospect” instances from a vast number of past reports.

## LLMs and certification exams

The full texts of questions from the national medical licensing exam and specialty board exams are, in most cases, publicly available, and extensive research has been conducted using LLMs to process these questions. The primary reason for this is that it allows for a rough assessment of whether AI can make clinical decisions. However, it is important to understand that there is a significant gap between solving exam questions and performing actual clinical imaging interpretation. When GPT-4 was used to answer Japanese national medical licensing examination questions, it outperformed GPT-3.5, suggesting that LLMs are being rapidly improved [63]. GPT-4 also performed well when tested with the Japanese diagnostic radiology board exam [64]. Oura and colleagues conducted an experiment shortly after the release of GPT-4, using board exams of Japanese diagnostic radiology, nuclear medicine, and interventional radiology (JDR, JNM, and JIR, respectively), where the accuracy rate was approximately 30–60%, indicating that there is still room for improvement [54]. Additionally, questions involving images tended to have lower accuracy rates. Hirano and colleagues found that in the JDR board exam, GPT-4 Turbo with Vision did not surpass the text-only version of GPT-4 Turbo [65]. These reports indicate that AI has still room for improvement once it comes to the task of converting images to language. Similar trials have been conducted with foreign specialty board exams, such as using Llama 3 to answer the American radiology board exam [66]. There have also been studies where GPT-4 was used to answer questions from the European board of radiology examinations [67, 68]. Research has also been done using the Polish specialty board exam as a subject [69], and there is a case where GPT-4 successfully answered the AJNR Journal’s Case of the Month [70]. In a study using Radiology Diagnosis Please Cases, the respective diagnostic accuracies of GPT-4o, Claude 3 Opus, and Gemini 1.5 Pro for primary diagnosis were 41.0%, 54.0%, and 33.9%, respectively [71].

## Utilization of LLMs in research

Radiology reports contain a wealth of case information and the accumulated knowledge of experts—a comprehensive form of "wisdom." The fundamental idea behind data mining is that by analyzing this information in detail, we may uncover medical insights that humans have not previously recognized. However, before the advent of LLMs, it was extremely challenging for computers to automatically perform statistical analyses on the knowledge contained within natural language texts. In the past, statistical models such as n-grams, hidden Markov models, recurrent neural networks (RNNs), and long short-term memory (LSTM) had been used; however, with the introduction of transformers and the practical application of LLMs, the structuring of text written in natural language has improved dramatically. One of the advantages of structuring texts is the ability to efficiently identify eligible cases from vast numbers of reports for inclusion in clinical trials. Doi and colleagues developed a DL-based NLP model that classifies the status of bone metastasis (BM) in radiology reports, including CT, MRI, and nuclear medicine, to detect patients with BM, and reported that the accuracy was about 0.9 [72]. Fink et al. attempted to structure CT reports of lung cancer and published the results of comparing several LLMs [73]. Paraneoplastic dermatoses [74] are an important condition, but due to their rarity, it is challenging to analyze a significant number of cases. By searching through a vast number of hospital reports using LLMs and conducting multi-center studies, it may be possible to build evidence for such rare conditions. Furthermore, LLMs have also been shown to be useful in the process of writing and reviewing academic papers [7]. Note that there is variability in how academic journals are addressing the use of LLMs; only 44% have policies regarding their use, with 43% applying these to authors and 30% to peer reviewers [75].

## Issues surrounding LLMs

The deployment of LLMs in various fields has highlighted several critical issues that need to be addressed. In particular, healthcare applications require exceptionally high levels of reliability. One key aspect of reliability is explainability [76]. Since LLMs often operate in ways that are opaque, it can be challenging for users to understand how conclusions are reached. Privacy is another major issue, as the vast amounts of data processed by LLMs can potentially expose sensitive information. Hallucinations, a phenomenon where the model generates incorrect or nonsensical results, are also a significant issue, as they can lead to serious consequences in clinical settings [77]. Bias in LLMs, particularly regarding race, gender, and other social factors, remains a significant challenge that can lead to unfair outcomes [78]. To improve accuracy and reliability, ongoing efforts are needed to refine these models and reduce such biases. The cost of cloud computing resources required to run LLMs is another barrier, limiting access to only well-funded institutions [79].

As LLMs continue to evolve, studies comparing models like ChatGPT, Claude, and Gemini are abundant, but it is essential to keep in mind that the superiority of one model over others is often short-lived as newer versions rapidly emerge [64, 80–83]. Consequently, when reviewing the older literature, it is crucial to consider the context of the technological landscape at the time of publication.

## Discussion

As we have observed, the number of papers on LLMs has been steadily increasing. This surge in research is largely due to the significant advantages of cloud-based AI, which makes LLMs more accessible and easier to study, drawing many researchers into the field. Another factor contributing to this trend is that researchers working on LLMs leverage these models to expedite paper production, benefiting from the efficiencies inherent in the technology.

**Fig. 4:**
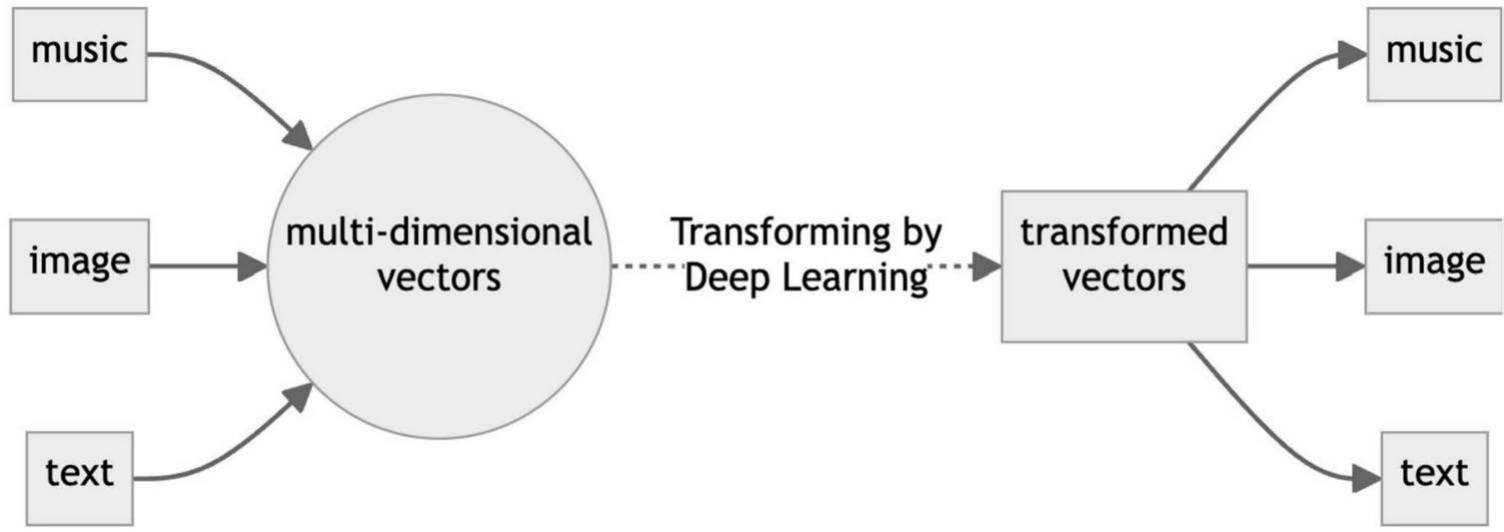
Adapted from Nakaura et al. 2024 [1]*.* Conceptual diagram illustrating the deep learning process. Data from different domains, such as music, image, and text, are input, and a variety of domains can be produced as output

**Fig. 5:**
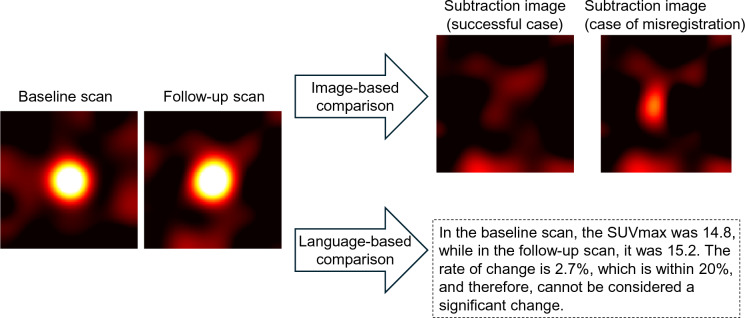
Simulation of tumor PET images. When evaluating treatment effects using baseline and follow-up scans, an image-based method involves aligning the images and performing subtraction. In cases with no actual change, a successful alignment will result in the tumor signal being cleanly removed. However, if there is misregistration between the two images, residual signal may persist, potentially leading to an inaccurate assessment of treatment effects. On the other hand, translating from the image domain to the language domain eliminates concerns about misregistration, enabling a more accurate and fair comparison of crucial information

AI, in a different phrasing, can be described as filling in the sparse parts within a space. The study of reducing radiation doses or shortening scanning time can be seen as a method of filling sparse data using statistical methods. Generative AI involves filling vast unknown areas from sparsely distributed known data, while traditional machine learning might only have filled one or two points, which did not seem very "generative." Generative AI is intriguing, because it applies not only within the same domain but also across different modalities like images, language, and audio data (Fig. 4). For computers, both text and images are represented as binary data (0 s and 1 s), and there is not a significant fundamental difference between them. The ability to convert information from the image domain into the language domain is particularly valuable in monitoring treatment response (Fig. 5). When judging treatment efficacy (e.g., chemotherapy, radiotherapy) with image processing such as image subtraction, aligning images (registration) is crucial, where even a few pixel misalignment can significantly affect the results. However, once information such as location and size is extracted from images and converted into language data, it becomes more resilient to minor discrepancies—much like how, in nuclear medicine, a one-pixel shift of region of interest (ROI) in measuring tumoral SUV does not alter the value of SUVmax.

While comparing treatment effects over time for the same patient is important, comparing images from different individuals is also of great interest. Such comparisons definitely require anatomical standardization if image-based comparison is intended, which can be quite challenging when only using image data. However, converting the image into natural language could potentially simplify the process. While it is true that some information is lost when converting from images to natural language, the information density per byte drastically increases, resulting in a highly compressed and rich dataset. If we can effectively compare data across different individuals, it could lead to the creation of normal databases which will be useful for establishing normal-database-based diagnosis. This concept helps account for variations due to race, gender, and age, potentially offering a more comprehensive understanding of human differences.

Unfortunately, a common sentiment among Japanese radiologists who do not regularly engage in nuclear medicine is a reluctance or lack of confidence regarding nuclear medicine exams due to their diversity. While medical exams like FDG-PET/CT or bone scintigraphy, which are performed frequently, are more familiar, other types of exams are seen less often. This infrequent exposure makes it challenging for less-experienced radiologists when writing a report. Additionally, there are concerns about the poor spatial resolution of some imaging techniques, making interpretation difficult. In this context, how can LLMs be beneficial? It is likely that LLMs will soon be able to find appropriate diagnostic names based on the similarity between typical images and those of current cases. However, what is needed in nuclear medicine diagnosis is not just arriving at a diagnostic name but understanding the pathological state. For example, if increased accumulation is seen in a bone scintigraphy, it indicates elevated bone metabolism, but whether this signifies bone metastasis or trauma requires a comprehensive evaluation. What is expected next is the integration of generative AI into this comprehensive diagnostic process. The impressive capability of generative AI lies in its ability to perform as if it were fine-tuned with minimal input from the user, whether through zero-shot, one-shot, or few-shot learning. The feasibility of applying such generative AI in nuclear medicine will need to be validated.

The validation of generative AI capabilities presents some challenges. A commonly used method, as mentioned earlier, is to test these models using exams such as medical licensing or board certification. However, there is a significant gap between solving these exams and performing actual image interpretation. Specialist exams often involve multiple-choice questions where hints to the answers can be found in the choice of text, even if the exact diagnosis is unknown. Therefore, to truly determine the utility of generative AI in nuclear medicine report generation, prospective studies where actual images are input and the quality of the generated reports is evaluated by humans are necessary.

At this point, it is also important to question whether reports are truly necessary. Additionally, one might ask whether images themselves are needed. The discussion about reports and images often revolves around the concept of explainability in AI. If explainability is not required, it might be sufficient for AI to propose treatment methods with no intermediate outputs, provided that the AI achieves a success rate of 99.9% or higher. However, because the performance of current AI is not that high and there is a possibility of errors in AI-generated answers, it is essential to comprehensively review each output result by human. This suggests that, without explainability, AI's clinical adoption might be limited. On the other hand, it is also noted that improving explainability may reduce accuracy (i.e., there is a trade-off between explainability and accuracy). The balance between these aspects will likely be a topic of future discussion.

Our approach to writing papers has also evolved by LLM. For instance, one of our authors now creates fragmented text by speaking into a smartphone while walking, generating very rough drafts. After that, the text is refined with the help of LLMs, eventually producing a polished and well-presented paper. The way we use our time has changed. Traditionally, work was done at a desk with the idea that it required individual effort. However, this view is changing. When it comes to filling in sparse information, there is no longer a need for human to do it manually; LLMs can generate content based on statistical calculations.

Even when faced with sparse data that seems impossible to complete, extensive past statistical data allow for multi-dimensional estimations of likely outcomes in similar cases. What is fascinating is that, despite generative AI only performing this task, it appears to possess a form of human-like intelligence. It suggests that a significant part of human intelligence might involve filling in sparse spaces. Generative AI utilizes empirical methods to complete these tasks. One area where generative AI is still considered weak is reasoning, although recent reports suggest that it is starting to improve in this area.

Nuclear medicine consists of three main components: (1) the administration of radiopharmaceuticals, (2) image acquisition (hardware), and (3) image generation and analysis (software). The advancements in each of these areas contribute synergistically to healthcare and medicine. Currently, AI in nuclear medicine seems to be focused primarily on software; however, it is evident that there are potential applications for generative AI in drug development and equipment development as well. In drug development, the task involves identifying promising compounds from a vast number of candidates. AI may excel in curating information, especially when similar drugs or compounds have been reported in various studies. AI could potentially outperform humans in this curation process. Additionally, AI might be useful in detecting research misconduct, such as data fabrication or falsification. By identifying such issues, AI can help prevent the reliance on erroneous studies and avoid the scenario where research efforts based on incorrect data lead to wasted resources and time.

Nuclear medicine is often referred to as functional imaging or molecular imaging, in contrast to morphological imaging (e.g., CT and MRI). It visualizes functions that are not directly observable, revealing intricate cascades within systems like the endocrine and nervous systems. While these cascades are challenging to represent, converting images into diagrams or text could help organize relationships between image findings and known physiological and neurological knowledge. Despite several factors suggesting that nuclear medicine might benefit from LLMs, there is a relatively small number of LLM-related papers in this field. This scarcity may be attributed to several reasons. Historically, research in nuclear medicine has lagged behind CT and MRI in terms of deep learning-based image analysis. Nuclear medicine excels in quantitative methods and often uses biologically meaningful metrics like SUVmax, which provide high informational content with simple indicators. This might explain why, despite the deep learning boom in image diagnostics since around 2015, nuclear medicine has not seen as much progress in deep learning. As another factor, the market for nuclear medicine is smaller compared to CT/MRI. Like other areas in radiology, nuclear medicine faces challenges with standardization and harmonization. Findings deemed useful in one study may not be applicable in other facilities, an issue that may persist even with the advent of LLMs. Nuclear medicine is well suited for deductive approaches, as opposed to purely machine learning-based methods.

In both writing and peer-reviewing papers, LLMs have become powerful tools. However, it is crucial to adhere to the policies set by various journals.

LLMs evolve extremely rapidly due to the competition among multiple models. While this evolution is beneficial for users, it is challenging to keep track of which model excels at which task. Given that these models are constantly changing, knowledge learned at one point may become outdated quickly. In the future, we may need a meta-model, which would continuously monitor the performance of the latest models and seamlessly select the model that best fits the user's needs at any given time.

There is ongoing debate about whether AI will replace human jobs and what humans should do if their roles are diminished by AI. Significant problems with AI, such as hallucinations, mean that no AI is currently allowed to handle all tasks entirely. There will always be aspects of medical decision-making that require human judgment. Therefore, it is crucial for us to continue training to make clinical decisions independently of AI. One interesting use of generative AI is to ask questions without fear, as the respondent is a machine rather than a person. For beginners, solving uncertainties is crucial, but the fear of appearing ignorant can hinder learning. Previously, learners had to rely on textbooks or, later, Internet search engines to address their questions. Now, we are approaching an era where generative AI can provide explanations based on its knowledge. In today’s world, where acquiring new knowledge is a major part of our work, generative AI will be useful across all levels of education, from undergraduate and graduate education to specialist training and continuing education later in life.

## Conclusion

In this review, we have investigated the latest insights into both LLMs and nuclear medicine to predict how LLMs might influence the future of nuclear medicine. While there are currently few direct applications of LLMs in nuclear medicine, it is anticipated that their use will increase in the future. LLMs hold significant potential to transform the existing frameworks in nuclear medicine, making this an area worth monitoring closely. Finally, this manuscript has been written as part of the activities of TOP GUN [84].
