# Correction: Generative AI and large language models in nuclear medicine: current status and future prospects

**DOI:** 10.1007/s12149-025-02024-9

**Published:** 2025-02-11

**Authors:** Kenji Hirata, Yusuke Matsui, Akira Yamada, Tomoyuki Fujioka, Masahiro Yanagawa, Takeshi Nakaura, Rintaro Ito, Daiju Ueda, Shohei Fujita, Fuminari Tatsugami, Yasutaka Fushimi, Takahiro Tsuboyama, Koji Kamagata, Taiki Nozaki, Noriyuki Fujima, Mariko Kawamura, Shinji Naganawa

**Affiliations:** 1 Department of Diagnostic Imaging, Graduate School of Medicine, Hokkaido University, Kita 15, Nishi 7, Kita-Ku, Sapporo, Hokkaido, 060-8638 Japan; 2 Department of Radiology, Faculty of Medicine, Dentistry and Pharmaceutical Sciences, Okayama University, Kita-Ku, Okayama, Japan; 3 Medical Data Science Course, Shinshu University School of Medicine, Matsumoto, Nagano Japan; 4 Department of Diagnostic Radiology, Tokyo Medical and Dental University, Bunkyo-Ku, Tokyo, Japan; 5 Department of Radiology, Osaka University Graduate School of Medicine, Suita-City, Osaka, Japan; 6 Department of Diagnostic Radiology, Kumamoto University Graduate School of Medicine, Chuo-Ku, Kumamoto, Japan; 7 Department of Radiology, Nagoya University Graduate School of Medicine, Showa-Ku, Nagoya, Japan; 8 Department of Artificial Intelligence, Graduate School of Medicine, Osaka Metropolitan University, Abeno-Ku, Osaka, Japan; 9 Department of Radiology, Graduate School of Medicine and Faculty of Medicine, The University of Tokyo, Bunkyo-Ku, Tokyo, Japan; 10 Department of Diagnostic Radiology, Hiroshima University, Minami-Ku, Hiroshima, Japan; 11 Department of Diagnostic Imaging and Nuclear Medicine, Kyoto University Graduate School of Medicine, Sakyoku, Kyoto Japan; 12 Department of Radiology, Kobe University Graduate School of Medicine, Chuo-Ku, Kobe, Japan; 13 Department of Radiology, Juntendo University Graduate School of Medicine, Bunkyo-Ku, Tokyo, Japan; 14 Department of Radiology, Keio University School of Medicine, Shinjuku-Ku, Tokyo, Japan; 15 Department of Diagnostic and Interventional Radiology, Hokkaido University Hospital, Kita-Ku, Sapporo, Japan

**Correction: Annals of Nuclear Medicine (2024) 38:853–864** 10.1007/s12149-024-01981-x

The article ‘Generative AI and large language models in nuclear medicine: current status and future prospects’, written by Kenji Hirata, Yusuke Matsui, Akira Yamada, Tomoyuki Fujioka, Masahiro Yanagawa, Takeshi Nakaura, Rintaro Ito, Daiju Ueda, Shohei Fujita, Fuminari Tatsugami, Yasutaka Fushimi, Takahiro Tsuboyama, Koji Kamagata, Taiki Nozaki, Noriyuki Fujima, Mariko Kawamura, Shinji Naganawa, was originally published under exclusive license to The Japanese Society of Nuclear Medicine. As a result of the subsequent decision to publish the article under the open access model, the article’s copyright notice was changed on 22 January 2025 to © The Author(s) 2025 and the article is now distributed under a Creative Commons Attribution 4.0 International License, which permits use, sharing, adaptation, distribution and reproduction in any medium or format, as long as you give appropriate credit to the original author(s) and the source, provide a link to the Creative Commons licence, and indicate if changes were made. The images or other third party material in this article are included in the article’s Creative Commons licence, unless indicated otherwise in a credit line to the material. If material is not included in the article’s Creative Commons licence and your intended use is not permitted by statutory regulation or exceeds the permitted use, you will need to obtain permission directly from the copyright holder. To view a copy of this licence, visit http://creativecommons.org/licenses/by/4.0/.

The original article has been corrected.

